# Superficial Abdominal Skin Laxity Versus Rectus Diastasis: Distinct Pathophysiological Entities With Different Functional and Reconstructive Implications in Modern Abdominoplasty

**DOI:** 10.7759/cureus.107427

**Published:** 2026-04-20

**Authors:** Andre Victor Baldin, Irvint Joel Bautista Perez, Mauricio Gonzalo Padilla Sierra, María Briceño-Suárez

**Affiliations:** 1 Plastic and Reconstructive Surgery, ABC Medical Center, Mexico City, MEX; 2 General Surgery, Universidad Nacional Autónoma de México, Mexico City, MEX; 3 General Surgery, Hospital General “Dr. Fernando Quiroz Gutiérrez”, Instituto de Seguridad y Servicios Sociales de los Trabajadores del Estado (ISSSTE), Mexico City, MEX; 4 General Practice, Universidad La Salle, Mexico City, MEX

**Keywords:** abdominal laxity, abdominal wall body contouring, abdominoplasty, fascial plication, linea alba, rectus diastasis

## Abstract

Abdominal contour deformities are commonly described as “abdominal flaccidity,” but they reflect distinct underlying anatomic components with different functional and surgical implications. This narrative review aimed to revisit the concept of abdominal flaccidity, propose a conceptual framework distinguishing cutaneous laxity from musculoaponeurotic weakness, clarify the anatomic and functional differences between superficial abdominal skin laxity and rectus diastasis, and discuss their implications for contemporary abdominoplasty. Indexed literature and seminal historical studies were synthesized to examine abdominal wall anatomy, imaging, post-pregnancy and post-weight loss deformities, fascial plication, mesh reinforcement, and current abdominoplasty strategies. The evidence supports that skin laxity primarily affects the cutaneous-subcutaneous envelope, whereas rectus diastasis reflects attenuation and widening of the linea alba with loss of midline tension, dynamic bulging, and, in selected patients, functional impairment. Contemporary abdominoplasty has evolved from dermolipectomy to individualized contour surgery integrating liposuction, selective undermining, vascular preservation, progressive tension concepts, and tailored management of the musculoaponeurotic layer. No single reconstructive technique is universally superior; however, phenotype-based evaluation improves indication, operative planning, perioperative safety, and outcome interpretation. Abdominal contour deformities should therefore be approached as a spectrum rather than a single entity in plastic surgery practice.

## Introduction and background

Abdominoplasty remains one of the most frequently performed aesthetic surgical procedures worldwide. In a 14-year analysis of International Society of Aesthetic Plastic Surgery data, more than 15.8 million surgical aesthetic procedures were reported globally in 2023, body and extremity procedures exceeded 5.1 million, and abdominoplasty consistently ranked among the five most performed aesthetic operations across the study period [1]. Contemporary certification data from the American Board of Plastic Surgery likewise confirm that abdominoplasty continues to occupy a central place in aesthetic practice, increasingly in the outpatient setting and within evolving safety algorithms [2,3].

Despite this procedural relevance, patients and surgeons still commonly use the umbrella term “abdominal flaccidity” to describe heterogeneous abnormalities of the abdominal contour. At minimum, two anatomical domains should be distinguished: (1) cutaneous laxity, defined by redundancy or poor recoil of the skin-subcutaneous envelope; and (2) musculoaponeurotic weakness, most commonly represented by rectus diastasis, which the European Hernia Society currently defines as a separation between the rectus muscles wider than 2 cm [4]. These domains often coexist, but they are not synonymous. Conflating them may blur indications for dermolipectomy, liposuction, flap undermining, fascial plication, or more formal abdominal wall reconstruction [4-6].

The epidemiology of the musculoaponeurotic component is better characterized than that of isolated skin laxity. In a prospective cohort of 300 primiparous women, Sperstad et al. reported rectus diastasis prevalences of 60.0% at six weeks postpartum, 45.4% at six months, and 32.6% at 12 months postpartum [7]. In a 2024 cross-sectional ultrasound study of 1,000 women assessed three to 30 years after childbirth, Lin et al. reported prevalence rates of 22-36% using an inter-rectus distance (IRD) threshold of >2 cm and 6-13% using a threshold of >3 cm [8]. These data underscore two important points: rectus diastasis remains common well beyond the puerperium, and reported prevalence is highly dependent on the diagnostic definition and measurement method used [7,8]. By contrast, population-based epidemiologic estimates for isolated abdominal skin laxity remain poorly standardized, which has contributed to persistent conceptual imprecision in both clinical discussion and operative planning.

Historically, abdominal contour surgery developed from procedures centered on skin and adipose excision toward progressively more analytical approaches to abdominal wall form. Vernon’s description of umbilical transposition in lipectomy and Pitanguy’s landmark 300-case series established essential foundations for modern abdominoplasty, whereas Regnault later systematized the historical evolution of abdominal dermolipectomy [9-11]. Decades later, Nahas reframed abdominal analysis around the myoaponeurotic layer, demonstrating that apparently similar abdominal contours may arise from fundamentally different structural deformities and therefore require different operative strategies [12].

Modern abdominoplasty and lipoabdominoplasty now integrate liposuction, selective undermining, preservation of vascular supply, and individualized correction of the musculoaponeurotic layer [13]. Contemporary imaging has further refined preoperative assessment by allowing objective characterization of IRD, its craniocaudal extent, associated ventral hernias, and dynamic wall behavior [14]. At the same time, recent reviews emphasize that the clinical significance of rectus diastasis, the optimal plication pattern, and the threshold for mesh reinforcement remain areas of heterogeneity and limited high-level evidence [15,16].

Accordingly, the purpose of this narrative review is to revisit the concept of abdominal flaccidity, distinguish cutaneous laxity from musculoaponeurotic weakness, clarify the anatomical and functional differences between superficial abdominal skin laxity and rectus diastasis, and propose a conceptual framework for surgical decision-making in contemporary abdominoplasty.

## Review

Methodology

This article was designed as a narrative review aimed at providing a conceptually focused and clinically applicable synthesis of the literature on abdominal contour deformities, with particular emphasis on distinguishing cutaneous laxity from musculoaponeurotic weakness/rectus diastasis in the context of contemporary abdominoplasty. A narrative review design was selected because the topic spans heterogeneous domains (including historical evolution, surgical anatomy, imaging, clinical assessment, functional implications, and operative techniques) that are not adequately addressed through a single narrowly framed systematic question. To improve transparency, coherence, and editorial quality, the review was structured according to the principles of the Scale for the Assessment of Narrative Review Articles (SANRA) [17], with explicit attention to the clinical relevance of the topic, the definition of the review aims, the transparency of the search strategy, the appropriateness of referencing, the consistency of scientific reasoning, and the presentation of clinically relevant data.

A targeted literature search was performed in PubMed/MEDLINE, Embase, Scopus, and Web of Science for articles published through March 2026. The search strategy combined controlled vocabulary and free-text terms related to the main conceptual domains of the review, including abdominoplasty, lipoabdominoplasty, abdominal contour deformity, abdominal flaccidity, skin laxity, cutaneous laxity, rectus diastasis, diastasis recti abdominis, linea alba, musculoaponeurotic layer, rectus sheath plication, fascial reconstruction, mesh repair, endoscopic repair, and post-bariatric body contouring. The search was limited to indexed medical literature in English or Spanish. Preference was given to recent publications from the last five years with a verifiable DOI, particularly systematic reviews, meta-analyses, clinical guidelines, consensus statements, randomized clinical trials, comparative studies, and prospective cohorts. Because the present review also sought to revisit the conceptual evolution of abdominal contour surgery, seminal historical articles were included irrespective of publication year when they were considered indispensable to contextualize the origin of dermolipectomy, belt lipectomy, myoaponeurotic analysis, classification systems, or foundational plication techniques.

Studies were considered eligible if they addressed at least one of the following domains: (1) historical development of abdominal contour surgery; (2) anatomy or imaging of the anterior abdominal wall and linea alba; (3) clinical or imaging-based characterization of rectus diastasis; (4) isolated skin laxity or post-weight loss abdominal skin redundancy; (5) fascial plication or reinforcement techniques; (6) contemporary concepts in abdominoplasty or lipoabdominoplasty; or (7) outcomes relevant to surgical decision-making, including recurrence, complications, function, quality of life, and patient-reported satisfaction. Nonindexed publications, duplicate reports, conference abstracts without full text, nonmedical sources, and articles lacking sufficient methodological or clinical detail for critical interpretation were excluded. Narrative opinion pieces without original data were not used as primary evidence unless they were historically foundational to the development of the field.

Titles and abstracts were screened for relevance, followed by full-text review of potentially eligible articles. Reference lists of key studies and reviews were hand-searched to identify additional relevant publications. For each included study, the following information was extracted when available: first author, year, country or setting, study design, population and selection criteria, sample size, principal dependent and independent variables, diagnostic criteria or measurement method, surgical technique, length of follow-up, main results, complications, and measures of association or effect. Given the heterogeneity of designs, populations, definitions of rectus diastasis, outcome measures, and operative techniques, no quantitative pooled analysis was attempted. Instead, the evidence was synthesized narratively and organized into thematic sections to support a component-based conceptual framework for surgical decision-making in abdominal contour deformities.

Historical evolution of abdominal contour surgery

Abdominal contour surgery did not evolve as a single operation, but rather as a progressive shift from functional excision of redundant tissue to anatomically tailored restoration of trunk contour. González-Ulloa’s description of belt lipectomy in 1960 expanded the operative field from isolated anterior abdominal tissue excision to circumferential truncal correction, establishing that deformity after pregnancy or major weight fluctuation could not always be adequately treated by simple lower abdominal dermolipectomy alone [18]. Lockwood’s high-lateral-tension abdominoplasty in 1995 further reframed the procedure around lateral tension vectors, waistline definition, and continuity between the abdomen, flanks, and thighs, rather than central skin redraping alone [19].

These developments reflected not only technical refinement but also a change in deformity analysis. In 2003, Aly et al. reported a retrospective single-center clinical series from the University of Iowa that included 32 patients with circumferential truncal excess undergoing belt lipectomy. The principal outcome was postoperative contour correction of the trunk as a unit, and morbidity was assessed from perioperative and postoperative records. Seroma occurred in 12 of 32 patients (37.5%), pulmonary embolism in three of 32 (9.3%), and one patient (3.1%) required reoperation for wound dehiscence. Despite this morbidity profile, the procedure improved not only the anterior abdomen but also mons ptosis, back rolls, waist definition, and buttock contour, reinforcing the concept that the trunk should often be treated as a three-dimensional aesthetic unit rather than as an isolated abdominal problem [20].

Attention later shifted from skin excision alone to deliberate correction of the musculoaponeurotic layer. In 2004, Yousif et al. compared six consecutive patients treated with transverse rectus sheath plication and bilateral crescent plication of the external oblique fascia with a similar group treated with standard vertical plication. This comparative observational study assessed the dependent variable through blinded postoperative photographic analysis by two impartial judges. The transverse plication group showed higher global scores (4.5 vs 3.9, p = 0.044), better anterior abdominal contour (4.7 vs 4.2, p = 0.029), and greater definition of the linea semilunaris (4.6 vs 3.7, p = 0.008). Differences in waistline contour (4.5 vs 3.8, p = 0.067), linea alba definition (4.4 vs 3.9, p = 0.067), and hip-waist transition (4.4 vs 3.7, p = 0.067) did not reach statistical significance [21].

Alternative plication geometries further emphasized that abdominal contour is influenced by vector redistribution and not only by midline narrowing. Marques et al. described 11 patients undergoing abdominoplasty with two fusiform plications placed at the transition between the rectus sheaths and the external obliques, aiming to create a narrower waist than that achieved by classical median xiphopubic plication [22]. Abramo et al. subsequently proposed H-shaped, double-contour plication, designed to reduce both longitudinal and transverse abdominal diameters and to improve cutaneous flap accommodation, illustrating the increasing technical interest in matching the plication pattern to the geometry of the deformity [23].

Contemporary lipoabdominoplasty represents the most mature expression of this evolution. In 2024, Saldanha et al. updated lipoabdominoplasty with an anatomical definition, emphasizing full liposuction, selective undermining, perforator preservation, Scarpa fascia suspension, and strict patient selection as the basis for integrating contour enhancement with reproducibility and acceptable morbidity [24]. Taken together, the historical trajectory of abdominal contour surgery shows a clear transition from tissue subtraction to component-specific management of the abdominal envelope, adipose layer, and musculoaponeurotic support.

Anatomy of the abdominal wall

For surgical decision-making, the anterior abdominal wall should be interpreted as two interacting but nonequivalent domains: the superficial cutaneous-subcutaneous envelope and the deep musculoaponeurotic layer. The abdominal wall includes skin, subcutaneous tissue, superficial fascia, the musculoaponeurotic complex, and deeper fascial-peritoneal structures. The rectus abdominis muscles are enclosed within the rectus sheath, which is formed by the aponeurotic contributions of the external oblique, internal oblique, and transversus abdominis muscles. At the midline, these fibers interlace to form the linea alba, the structure most directly implicated in rectus diastasis [25].

In a 2001 anatomical study, Axer et al. examined the collagen architecture of the linea alba and rectus sheaths and described the midline not as a simple linear raphe, but as a three-dimensional, highly organized collagen meshwork. This finding is important because it explains why rectus diastasis is usually expressed as widening and attenuation of the linea alba rather than as a discrete fascial hole [25].

Normative imaging studies have clarified how variable the linea alba can be in women without prior pregnancy. In 2023, Woxnerud et al. conducted a cross-sectional ultrasound study in Stockholm, including 71 healthy nulliparous women. The dependent variables were IRD and linea alba thickness, measured at standardized anatomical points; explanatory variables included age, BMI, waistline activation, and physical function questionnaires. The mean age was 30.5 years, and the mean BMI was 23.5 kg/m². The mean IRD was 10 mm at the superior umbilical border, 9 mm at 3 cm above the umbilicus, and 2 mm at 2 cm below the umbilicus, while the mean linea alba thickness was 3 mm. These data provide a practical anatomical reference for the nonpostpartum abdominal wall rather than an operative threshold by themselves [26].

In 2022, Kaufmann et al. performed a retrospective cross-sectional CT study of 329 asymptomatic adults evaluated between 2016 and 2018. The dependent variable was IRD at predefined abdominal levels, and the principal independent variables were age, BMI, and parity. Using the prevailing definition of diastasis recti abdominis, the authors found a prevalence of 57%. At 3 cm above the umbilicus, the median IRD was 22 mm, and the 80th percentile was 34 mm. Age, BMI, and parity were statistically significant risk factors for wider linea alba measurements. These findings suggest that a fixed universal threshold may overclassify anatomical widening as surgical disease if context is ignored [27].

In parallel, Tuominen et al. reported a prospective epidemiological cohort study in Helsinki in which women were recruited during early-pregnancy ultrasound from January 1, 2018 to March 8, 2019. The main dependent variable was sonographic linea alba width/IRD, while symptoms were measured by questionnaire using RAND-36 and the Oswestry Disability Index. Among 933 women, mean IRD was 1.81 ± 0.72 cm in nulliparous participants, 2.36 ± 0.83 cm after one previous pregnancy, and 2.55 ± 1.09 cm after more than one pregnancy. Previous pregnancies correlated positively with increased linea alba width (p = 0.00004), whereas severe diastasis greater than 5 cm was uncommon [28].

Accordingly, rectus diastasis should be understood as widening and attenuation of the linea alba with lateralization of the rectus muscles, not as a true ventral hernia. Swedish national recommendations similarly describe diastasis as a condition commonly associated with midline bulging without fascial defect, recommend imaging when concomitant umbilical or epigastric hernia cannot be excluded, and consider linea alba plication the first-line surgical technique when operative treatment is indicated [29]. This distinction is critical because neither the presence of superficial skin redundancy nor the measured IRD alone fully defines the dominant anatomical problem requiring correction.

Skin laxity as an isolated component of abdominal flaccidity

**Figure 1:**
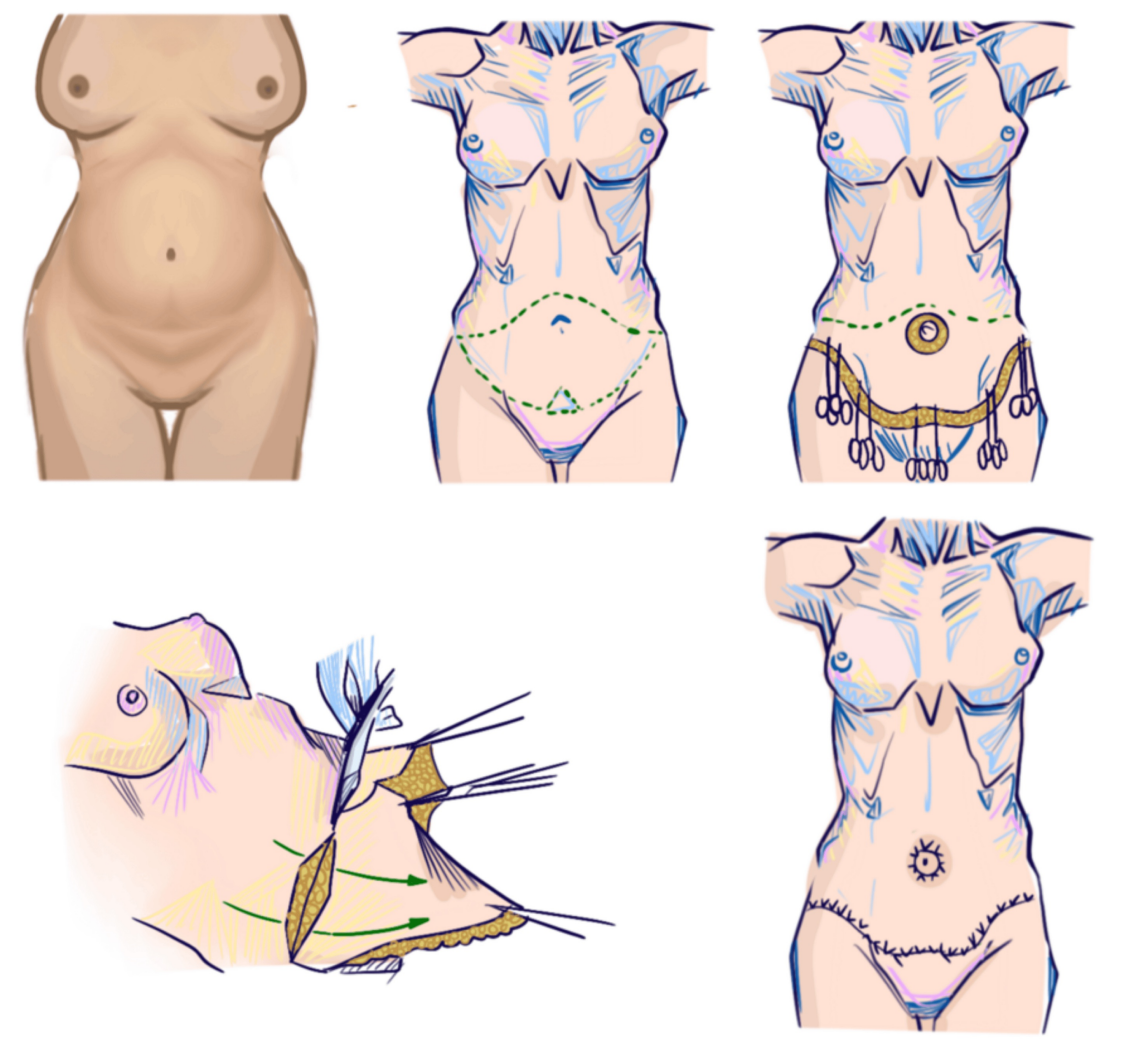
Illustration of superficial abdominal laxity and its correction with isolated dermolipectomy This figure illustrates the surgical management of superficial abdominal laxity characterized by redundant skin and subcutaneous tissue without significant musculoaponeurotic weakness. The sequence depicts the preoperative appearance, planned skin excision, isolated dermolipectomy with cutaneous-subcutaneous resection, flap redraping, and final closure without fascial plication. Artwork created by Irvint Joel Bautista Perez using the Procreate app (Savage Interactive Pty Ltd, Hobart, Australia).

Skin laxity is a disorder of the abdominal envelope rather than of the midline aponeurosis. Its clinical expression includes decreased recoil, infraumbilical redundancy, striae-bearing skin, hanging folds, and pannicular descent. These findings may persist despite preserved anterior wall support or only minimal inter-rectus widening. In this phenotype, the dominant deformity is superficial: excess skin and subcutaneous tissue distort abdominal contour even when musculoaponeurotic weakness is absent or not the principal driver of protrusion (Figure 1).

The post-bariatric population illustrates this concept particularly well. Song et al. developed and validated the Pittsburgh Rating Scale as a structured classification of contour deformities after bariatric weight loss. In that validation study, agreement across body regions was at least substantial, with kappa coefficients ≥0.60, a mean kappa of 0.68, and overall agreement of 69%, providing a reproducible framework for grading post-weight loss skin redundancy and deformity severity [30].

In 2019, Monpellier et al. studied post-bariatric patients selected from a prospective Dutch database using electronic questionnaires that evaluated demographics, overhanging skin, body satisfaction, and desire for body contouring surgery. Among 590 patients, 368 (62.4%) desired body contouring surgery, 157 (26.6%) did not, and 65 (11.0%) had already undergone it. Patients who desired surgery had more body areas affected by overhanging skin and more frequently scored Pittsburgh grade 3 than those without such a desire. In addition, 39.1% of the “desire” group had never consulted a plastic surgeon, and 44.1% of these patients met the weight criteria for reimbursement. These data indicate that skin redundancy after massive weight loss is not a minor aesthetic detail but a measurable, multiregional burden with functional, psychological, and access-to-care implications [31].

From a reconstructive perspective, skin laxity after massive weight loss is rarely limited to a simple lower abdominal pannus. Herman et al. emphasized that excess skin in this population often involves the abdomen, flanks, mons, back, and lower trunk simultaneously, which explains why some patients are not adequately treated by isolated low-transverse dermolipectomy and instead require extended abdominoplasty, fleur-de-lis patterns, or circumferential procedures according to deformity distribution [32].

At the tissue level, Hany et al. in 2024 conducted a prospective histological comparison of skin changes in post-bariatric and nonbariatric massive weight loss patients using 80 biopsies obtained during body-contouring procedures. Ultimately, 77 patients were analyzed (38 surgical massive weight loss and 39 nonsurgical massive weight loss). In abdominal samples, dermal elastic fibers were lower in the surgical massive weight loss group than in the nonsurgical massive weight loss group (p = 0.029), whereas abdominal complication rates after abdominoplasty did not differ significantly (p = 1.000). These findings support the clinical impression that cutaneous laxity is not merely a matter of surface excess; it may also reflect altered dermal quality [33].

When physical examination demonstrates minimal midline separation, preserved trunk support, and a deformity dominated by skin envelope excess, the operative strategy should prioritize skin and subcutaneous redraping rather than routine fascial tightening. Conversely, in post-weight loss patients with a broader distribution of excess tissue, the superficial problem may extend beyond the central abdomen and require longer excisional patterns or circumferential approaches [30-33].

Musculoaponeurotic weakness and rectus diastasis

**Figure 2:**
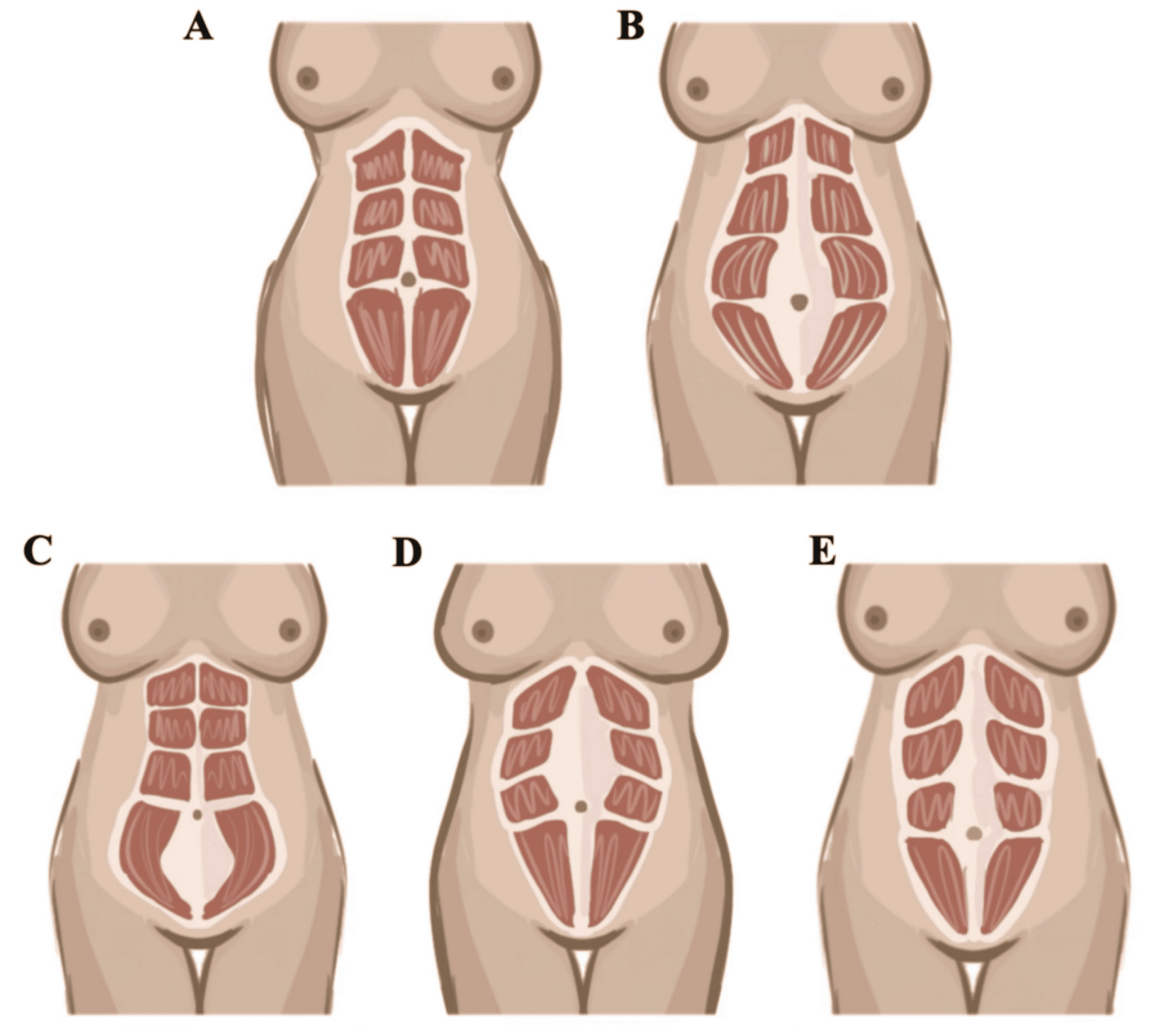
Patterns of rectus abdominis diastasis reflecting different distributions of linea alba weakness This figure illustrates different anatomical patterns of rectus diastasis as a manifestation of musculoaponeurotic abdominal wall weakness. (A) Normal abdomen without rectus diastasis. (B) Wide or global diastasis with separation of the rectus muscles along most of the linea alba. (C) Infraumbilical diastasis, predominantly affecting the lower abdominal segment. (D) Supraumbilical diastasis, confined mainly to the upper abdominal segment. (E) Complete diastasis involving both supraumbilical and infraumbilical regions. Artwork created by Irvint Joel Bautista Perez using the Procreate app (Savage Interactive Pty Ltd, Hobart, Australia).

Unlike isolated skin laxity, musculoaponeurotic weakness reflects a deformity of the deep abdominal wall support system. Its most recognizable manifestation is rectus diastasis, a condition in which the linea alba becomes widened and attenuated, allowing lateral displacement of the rectus muscles and loss of effective midline tension (Figure 2). A recent evidence-based review of the surgical treatment of rectus diastasis emphasized that the deformity should not be interpreted solely as a cosmetic widening of the midline, but rather as a structural alteration that may coexist with abdominal protrusion, trunk instability, discomfort, and reduced functional performance, depending on patient selection and diagnostic threshold [34].

The functional significance of rectus diastasis depends in part on how it is defined. In 2024, Wang et al. performed a retrospective cohort study of 437 long-term postpartum women recruited at three, five, 10, 20, and 30 years postpartum in China. The dependent variable was diastasis recti abdominis, measured by ultrasound IRD and by the presence of linea alba or umbilical hernia; main outcome variables included abdominal muscle strength, low back pain, urinary incontinence, and quality of life, assessed with standardized questionnaires. When diastasis was defined as IRD >2 cm, women with diastasis had more severe low back pain and poorer quality of life only at 10 years postpartum. When the threshold was raised to IRD >3 cm, women with diastasis showed weaker abdominal muscle strength, more severe low back pain at three, five, and 10 years postpartum, poorer quality of life at three, five, 10, and 20 years postpartum, and a higher incidence of linea alba or umbilical hernia at five and 20 years postpartum [35].

This threshold sensitivity is clinically relevant because not all widening of the linea alba should be interpreted as the same surgical entity. In 2024, Shen et al. proposed and clinically validated a width-length ultrasound classification for diastasis recti abdominis. In the pilot retrospective cohort, 301 patients were classified according to the width and craniocaudal extent of the separation; in the prospective validation cohort, 100 patients were enrolled, and treatment allocation was compared with the proposed classification. Type 1 accounted for 108 patients, Type 2 for 63, Type 3 for 44, Type 4 for 74, and Type 5 for 12. Types 1 and 2 were associated with conservative treatment recommendations, whereas Types 3-5 were associated with surgical treatment recommendations (all p < 0.05). In the validation cohort, the classification achieved 86.0% accuracy for guiding treatment strategy [36].

The most clinically relevant subgroup remains the symptomatic patient with persistent trunk dysfunction despite conservative treatment, rather than the patient with a widened linea alba alone. In 2019, Olsson et al. conducted a prospective cohort study in Stockholm that included 60 postpartum women with symptomatic diastasis recti abdominis and trunk instability resistant to training. The dependent variable was postoperative abdominal trunk function, measured with the Abdominal Trunk Function Protocol; secondary outcomes included recurrence by CT, urinary incontinence by UDI-6/IIQ-7, and quality of life by SF-36. At one year, there was no recurrence. Self-reported abdominal trunk function improved in 98% of patients, with a mean score improvement of 79.1%; 76% improved in physiotherapist-monitored functional tests; and all SF-36 subscales improved significantly vs baseline. Fewer urinary symptoms were reported by 47% on UDI-6 and 37% on IIQ-7, whereas 13% and 8%, respectively, reported worsening [37].

Surgical techniques for fascial reconstruction

Once musculoaponeurotic weakness has been identified as a dominant component of the deformity, fascial reconstruction becomes a central objective of surgery. Yet, despite the large number of published techniques, no single repair has emerged as a universal standard. In 2025, Tuominen et al. reported a single-center, superiority, double-blind randomized clinical trial from Finland that compared plication supported by mesh (PSUM) with suture plication alone in 86 normal-weight women with symptomatic postpartum rectus diastasis, of whom 84 (98%) completed follow-up. The dependent variable was recurrence at one year, defined as IRD >20 mm; the principal secondary outcomes were absolute IRD reduction, health-related quality of life, Oswestry Disability Index, motor control, and complications. Recurrence occurred in two of 44 (5%) PSUM patients and two of 40 (5%) suture plication patients (p = 0.922). Mean IRD reduction was greater with PSUM (52 mm; 95% CI 48-56) than with suture plication (44 mm; 95% CI 40-47; p < 0.002). Both groups improved in health-related quality of life, low back disability, and motor control (p = 0.000-0.039), and complication rates did not differ (17 of 44 (39%) vs 13 of 40 (33%); p = 0.558) [38].

This randomized evidence should be interpreted alongside broader pooled evidence. In 2021, Van Kerckhoven et al. performed a systematic review of rectus diastasis treatment that included 24 studies selected from 53 eligible publications, representing 931 surgically treated patients. Primary outcomes were recurrence and perioperative complications; secondary outcomes included satisfaction, chronic pain, and quality of life. Across the pooled sample, recurrence was reported in 5%, seroma in 7%, abdominal hypoesthesia in 6%, surgical site infection in 2%, and chronic pain in 4%. Follow-up ranged from three weeks to 20 years. The authors concluded that durability, safety, and patient satisfaction supported surgical correction of rectus diastasis, but the evidence did not favor plication over routine mesh reinforcement or vice versa [39].

Newer reinforcement strategies have attempted to preserve the technical flexibility of suture repair while improving resistance to pull-through. In 2024, Marangi et al. reported a prospective comparative study in which 65 of 70 initially treated patients with rectus abdominis diastasis completed six months of follow-up; 33 patients underwent repair with a suturable polypropylene mesh (Duramesh), and 32 underwent standard 0 polypropylene suture plication. The dependent variables included safety endpoints (infection, seroma, hematoma, wound dehiscence, fistula, and hospital stay), recurrence by ultrasound, palpability of the repair, postoperative pain by Visual Analogue Scale (VAS), and patient satisfaction by BODY-Q. No significant differences were observed between groups for infection, seroma, hematoma, wound dehiscence, fistula, hospital stay, VAS, or BODY-Q. The mesh-based device reduced the time required to perform plication compared with detached polypropylene sutures [40].

At the other end of the technical spectrum, long-lasting absorbable suture plication remains a defensible option in selected aesthetic patients. In 2024, Jackson et al. published a retrospective series of abdominoplasty patients treated between 2018 and 2022 by a single senior author, including only female patients with more than six months of follow-up. The dependent variable was clinical recurrence of rectus diastasis after plication with interrupted figure-of-eight polydioxanone and running Maxon sutures, assessed by physical examination at postoperative visits. Among 71 patients, mean follow-up was 21.1 months, mean age was 43 years, and mean BMI was 27 kg/m². No recurrence of diastasis was identified (0% recurrence rate). Complications included delayed wound healing in 11%, seroma in 8.5%, hematoma in 2.8%, and deep vein thrombosis/pulmonary embolism in 2.8%; no patient required reoperation [41].

Contemporary concepts in abdominoplasty

Contemporary abdominoplasty is no longer defined by skin excision alone. Instead, it combines deformity analysis, safe flap management, selective treatment of the musculoaponeurotic layer, and, when appropriate, liposuction or minimally invasive midline repair. In 2024, Medina et al. reported a multicenter comparative study from Rome and Buenos Aires that included 170 women with diastasis recti ≥2 cm, no previous abdominal procedures, and a minimum follow-up of 12 months. Group I included 85 consecutive patients treated through abdominoplasty access, whereas Group II included 85 consecutive patients treated through an endoscopic approach. The dependent variables were infection, seroma, hematoma, wound dehiscence, hospital stay, operative time, and recurrence detected by ultrasound sonography. Mean operative time was longer in the abdominoplasty group (176 minutes (145-201)) than in the endoscopic group (80 minutes (55-105); p < 0.05), whereas drain duration was shorter (2.8 vs 5.3 days; p < 0.05). Hospital stay did not differ (2 vs 1.5 days; p = 0.565). Infection occurred in 1.17% vs 2.35% (p = 0.347), seroma in 4.7% vs 7.05% (p = 0.182), hematoma in 4.7% vs 5.88% (p = 0.876), wound dehiscence in 3.52% vs 2.35% (p = 0.794), and recurrence in 2.35% vs 3.52% (p = 0.911) for abdominoplasty and endoscopic repair, respectively. These results support the view that open and minimally invasive approaches are both viable, but they serve different patient priorities [42].

Dead-space control has become another central concept in modern abdominoplasty. In 2024, Rao et al. published a systematic review and meta-analysis registered in PROSPERO (CRD42022346106) that included 24 studies and 750 patients, including two randomized controlled trials, comparing progressive tension suturing (PTS) with drains. The dependent outcomes were seroma, reoperation, hematoma, infection, and dehiscence. PTS significantly reduced seroma (RR 0.34; 95% CI 0.15-0.76; p = 0.001) and reoperation (RR 0.56; 95% CI 0.03-9.77; p = 0.05) vs drains, without significant differences in hematoma or infection. In subgroup analysis, the combination of liposuction + PTS was associated with lower seroma (RR 0.18; 95% CI 0.00-7.39; p < 0.00001), lower infection (RR 0.16; 95% CI 0.03-0.86; p = 0.03), and lower dehiscence (RR 0.11; 95% CI 0.01-1.01; p = 0.05) than drain-based strategies [43].

These pooled findings are consistent with larger contemporary single-center series. In 2024, Bendon et al. reported the United Kingdom experience with 286 consecutive patients undergoing drainless lipoabdominoplasty with a lipoaspirate volume of 500 mL or greater between 2017 and 2023. The dependent outcomes were all complications, delayed healing, seroma, and revision. The mean lipoaspirate volume was 2392.4 mL (range 500-5900 mL), and the mean abdominal tissue resection weight was 1392.0 g (range 346-3802 g). Minor local irregularities occurred in 14.0%, abdominal scar problems in 12.9%, umbilical shape/scar issues in 4.5%, localized infection in 4.2%, and delayed healing in 3.8%. There was one abdominal hematoma, one venous thromboembolism (0.3%), one drug-induced hepatitis (0.3%), and a seroma rate of 3.1%. Revision under general anesthesia was required in 16.0%, and there was no significant relationship between lipoaspirate volume and any of the four primary outcomes [44].

Proposed conceptual framework for surgical decision-making

A practical surgical framework should begin by abandoning the generic term “abdominal flaccidity” as a standalone diagnosis. Contemporary consensus work recommends a structured approach centered on accurate diagnosis, tailored treatment, and patient-centered decision-making, particularly in post-gravidic rectus abdominis diastasis. In practical terms, the surgeon should determine whether the dominant deformity lies in the superficial envelope, the musculoaponeurotic layer, or both. Patients with minimal skin excess, dynamic midline bulging, and functionally relevant diastasis may be candidates for isolated midline repair, including minimally invasive options. By contrast, when redundant skin, pannicular descent, striae-bearing lower abdominal envelope, or adipocutaneous excess are clinically relevant, the operation should address the envelope directly rather than relying on isolated deep repair alone [45].

**Figure 3:**
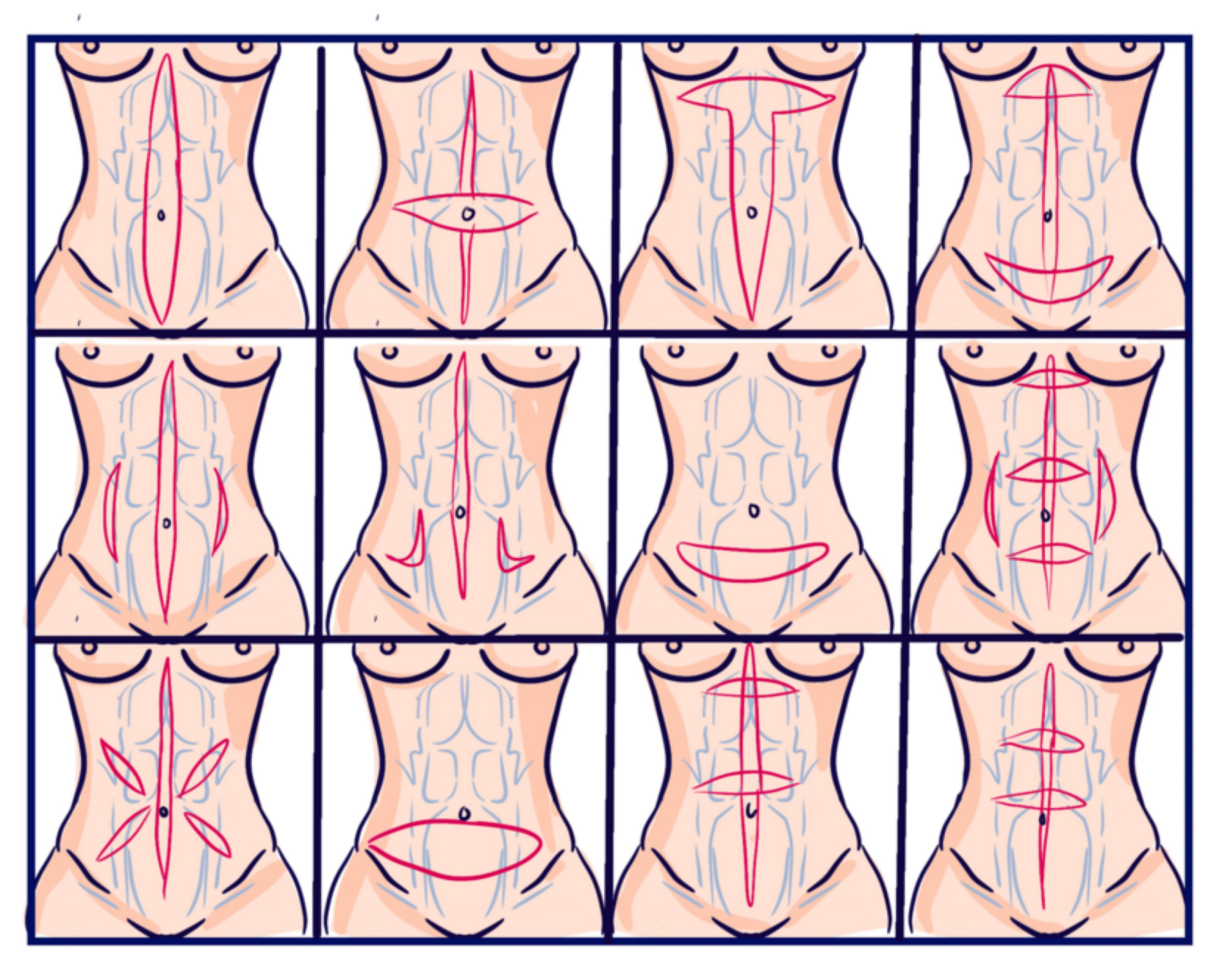
Abdominal wall plication patterns for rectus diastasis repair This figure illustrates representative abdominal wall plication configurations used in the management of rectus diastasis and musculoaponeurotic weakness. The depicted patterns reflect commonly described approaches in the surgical literature aimed at restoring midline tension and improving abdominal contour. Source references: [10,12,21-23] Artwork created by Irvint Joel Bautista Perez using the Procreate app (Savage Interactive Pty Ltd, Hobart, Australia).

When the predominant phenotype is musculoaponeurotic weakness with moderate or severe loss of abdominal wall tone, the surgeon should not assume that standard midline plication is always sufficient (Figure 3). In a retrospective cohort of 56 patients undergoing mesh abdominoplasty for moderate-to-severe rectus diastasis, Dumanian and Moradian reported a 0% surgical site occurrence rate, with 40 women and 16 men, superficial infections in three patients, and office soft-tissue revision rates of 23% in women and 6% in men. In a related comparative retrospective cohort, Sood et al. compared 40 patients undergoing retrorectus mesh repair with 37 patients undergoing standard suture plication. The primary endpoint was surgical site occurrence. Surgical site occurrence was 0% in the mesh group vs 19% in the standard group (p = 0.005); soft-tissue revision rates were 23% vs 27% (p = 0.84). Although the mesh group had lower preoperative aesthetic scores (65.8 ± 11.6 vs 70.3 ± 11.4; p = 0.0013), both groups improved significantly after surgery (mesh: 75.9 ± 12.6, p < 0.0001; suture: 82.5 ± 11.4, p < 0.0001). These data suggest that reinforcement may be particularly relevant in selected severe phenotypes, but not necessarily as a routine default for all diastasis repairs [46,47].

Discussion

The main contribution of this review is conceptual rather than purely technical: abdominal flaccidity should not be interpreted as a single deformity. Instead, it should be understood as a composite term that may reflect, in different proportions, a disorder of the superficial abdominal envelope (skin and subcutaneous tissue), a disorder of the deep musculoaponeurotic layer (most commonly rectus diastasis), or the coexistence of both. This distinction is not semantic. It directly affects indication, incision choice, the need for flap undermining, the relevance of fascial repair, and the balance between aesthetic and functional goals. Recent comprehensive reviews have shown that the literature on diastasis recti abdominis remains heterogeneous in terminology, diagnostic thresholds, symptom attribution, and outcome reporting, which helps explain why surgical decision-making still varies substantially across specialties and practice settings [48].

Our first objective was to revisit the concept of abdominal flaccidity. The literature reviewed here supports abandoning the routine use of this expression as if it denoted a uniform anatomical diagnosis. In clinical practice, patients may present with a protruding abdomen caused predominantly by deep midline failure, with relatively limited skin excess, whereas others may present with redundant, striae-bearing, poorly retractile skin and subcutaneous excess despite only minimal inter-rectus widening. These phenotypes are not equivalent, and they should not be expected to respond equally to the same operation. This distinction becomes even more relevant in postbariatric body contouring. In a multicenter prospective study including 305 postbariatric patients, Wilting et al. found that body contouring surgery improved health-related quality of life regardless of plication status, but the improvement was greater in the plication group for all domains and, after multivariable adjustment, remained significant for body image (p = 0.001), psychological function (p = 0.035), and sexual function (p = 0.035) [49]. These findings suggest that deep repair matters when the musculoaponeurotic component is clinically relevant, but not every patient with abdominal contour dissatisfaction necessarily requires rectus repair.

Our second and third objectives were to distinguish cutaneous laxity from musculoaponeurotic weakness and to clarify the anatomical and functional differences between superficial skin laxity and rectus diastasis. The evidence synthesized in the present review indicates that the most important discriminator is not simply the measured width of the linea alba, but the clinical phenotype that results from it. Rectus diastasis becomes surgically meaningful when widening of the linea alba is associated with dynamic bulging, loss of trunk support, dissatisfaction not explained by skin excess alone, or associated small midline hernias. In this context, the recent report by Saxen et al. is relevant because it moves the discussion beyond short-term technical success. In their long-term retrospective analysis of patients with post-pregnancy moderate-to-severe rectus diastasis and concomitant small primary midline hernias treated with HELP abdominoplasty, no recurrence of diastasis or hernias was reported after a mean follow-up of 5.2 years, and the overall complication rate was 11.8% [50]. This does not establish superiority of one technique over another, but it reinforces the idea that selected patients with clinically meaningful musculoaponeurotic failure can achieve durable benefit when the entire damaged midline is addressed as a structural problem rather than as a purely aesthetic concern.

Our fourth objective was to discuss the surgical implications of this distinction in contemporary abdominoplasty. The present review suggests that operative strategy should be phenotype-driven, not technique-driven. In other words, the surgeon should first determine whether the dominant problem is envelope excess, deep support failure, or both, and only then choose between isolated dermolipectomy, standard abdominoplasty with plication, extended or circumferential excision, or a minimally invasive approach to midline repair. The problem is that the available evidence still does not support a universally superior reconstructive method. Real-world nationwide data underline this uncertainty. In their 13-year analysis of German hospital discharge data, Paasch et al. identified 2,768 rectus diastasis repairs without concomitant hernia, with a mean of 197.7 procedures per year, mesh use in 28.0%, and an overall early complication rate of 6.9%. Importantly, the authors emphasized key limitations of administrative datasets, including coding bias, underreporting, and lack of information on surgical technique or specialty, and concluded that prospective registries are needed to standardize interpretation of outcomes [51]. This is precisely the type of heterogeneity that has historically obscured the difference between correcting a contour problem and reconstructing a structural one.

Contemporary abdominoplasty must also be interpreted through the lens of safety, because one of the practical implications of distinguishing skin laxity from musculoaponeurotic weakness is that it can prevent overtreatment. If a patient with isolated skin redundancy undergoes unnecessary aggressive fascial reconstruction, or if a patient with severe multidirectional post-weight loss deformity is offered only limited correction, the result may be technically successful yet conceptually mismatched (Table 1). The current trend toward outpatient aesthetic surgery further increases the importance of precise patient selection and preoperative optimization. In a retrospective chart review of 2,581 patients undergoing aesthetic surgery in a Canadian ambulatory surgery center, Ziolkowski et al. found abnormal bloodwork in 32.5%, abnormal electrocardiograms in 9.3%, and 242 patients (9.4%) with alterations in care resulting from preoperative workup, including additional investigations, specialist consultation, pharmacotherapy, and postponement of surgery [52]. Likewise, Rohrich et al., reviewing 42,720 consecutive outpatient plastic surgery cases over three decades, reported an overall complication rate of 0.74%, but demonstrated that higher BMI, longer operative duration, and combined procedures were associated with venous thromboembolism or inpatient transfer [53]. These data support a principle highly relevant to abdominoplasty: modern outcomes depend not only on operative design, but also on appropriate setting, risk stratification, and restraint.

**Table 1:** Conceptual distinction between cutaneous laxity and rectus diastasis in abdominal wall deformities This table summarizes the key clinical, anatomical, and surgical differences between cutaneous abdominal laxity and rectus diastasis, highlighting their distinct pathophysiological mechanisms and implications for surgical management. Table created by Irvint Joel Bautista Perez.

Characteristic	Cutaneous laxity (superficial component)	Rectus diastasis (deep component)
Definition	Redundancy and loss of elasticity of the skin and subcutaneous tissue	Separation of the rectus muscles due to widening of the linea alba
Anatomical level	Skin and subcutaneous tissue	Musculoaponeurotic system
Main etiology	Aging, pregnancy, weight loss, genetic factors	Pregnancy, increased intraabdominal pressure, fascial weakness
Clinical manifestation	Skin folds, striae, pannus, redundant skin	Dynamic bulging, central protrusion, core weakness
Functional impact	Limited, mainly aesthetic	May be associated with low back pain, core dysfunction, and reduced quality of life
Diagnostic evaluation	Clinical inspection	Dynamic examination + ultrasound or CT
Surgical implication	Dermolipectomy/superficial abdominoplasty	Rectus plication/linea alba reconstruction
Risk if left untreated	Low functional impact	High risk of persistent bulging

This issue becomes even more critical in patients after massive weight loss, a population in whom skin laxity is often the dominant complaint but not the only deformity present. In a large single-series comparative study of 196 patients undergoing abdominoplasty after bariatric weight loss, Cannistrà et al. reported an overall postoperative complication incidence of 12.6%, with seroma in 2.5%, partial dehiscence/skin necrosis in 2.0%, and a mean postoperative hospital stay of 3.6 days [54]. At a larger scale, Chaker et al. analyzed 55,596 abdominoplasty patients and found an overall complication rate of 2.1%, with significant differences between procedure types; notably, fleur-de-lis abdominoplasty had the highest complication rate, and male sex, diabetes, underweight status, and morbid obesity were independent risk factors for major complications [55]. These studies reinforce that the superficial deformity is not trivial in post-weight loss patients and that broader excisional strategies, while often appropriate, may carry a distinct morbidity profile. Therefore, our proposed framework should not be understood as a binary algorithm, but as a means of identifying the dominant anatomical driver of deformity while incorporating risk, extent of tissue excess, scar tolerance, and patient priorities.

**Figure 4:**
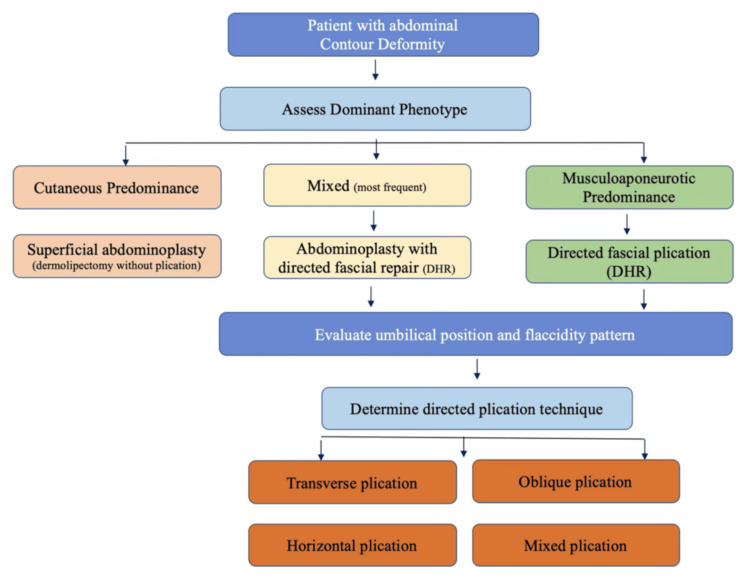
Phenotype-based algorithm for the surgical management of abdominal contour deformities This schematic algorithm illustrates a phenotype-based approach to the evaluation and surgical management of abdominal contour deformities. Patients are initially classified according to the predominant component of deformity as cutaneous, musculoaponeurotic, or mixed. Treatment strategies are then tailored accordingly, ranging from superficial abdominoplasty in cases of isolated cutaneous laxity to directed fascial repair (DHR) in patients with musculoaponeurotic weakness. In mixed cases, combined approaches are indicated. Final surgical planning is guided by the assessment of umbilical position and the distribution of abdominal flaccidity, allowing selection of the most appropriate plication pattern, including transverse, oblique, or combined configurations. Original illustration created by Irvint Joel Bautista Perez.

This review has limitations. By design, it is a narrative review, not a systematic review or meta-analysis. The available literature itself is limited by nonuniform definitions of diastasis, variable imaging protocols, inconsistent use of patient-reported outcome measures, and a predominance of retrospective or single-center studies. In addition, the historical development of abdominoplasty has been shaped by parallel contributions from aesthetic plastic surgery and abdominal wall reconstruction, two fields that do not always use the same language or prioritize the same endpoints [48-51]. Nevertheless, the present review offers a clinically useful synthesis: skin laxity and musculoaponeurotic weakness are distinct but interacting components of abdominal contour deformity, and contemporary abdominoplasty should be planned according to their relative contribution rather than according to habit, dogma, or terminology alone. In this sense, the proposed framework is not intended to replace surgeon judgment, but to make that judgment more anatomically explicit, more reproducible, and ultimately more aligned with the true source of deformity (Figure 4).

## Conclusions

Abdominal contour deformities should no longer be approached under the undifferentiated label of abdominal flaccidity. From a clinical and surgical standpoint, the critical step is to determine whether the dominant deformity lies in the cutaneous envelope, the musculoaponeurotic layer, or the coexistence of both. Skin laxity and rectus diastasis are anatomically and functionally distinct entities, and treating them as interchangeable findings may lead either to persistent postoperative bulging after adequate skin excision or to unnecessary fascial plication in patients whose deformity is primarily superficial.

In contemporary abdominoplasty, optimal outcomes depend on component-based evaluation, technical individualization, and standardized perioperative safety principles. A conceptual framework that distinguishes superficial skin redundancy from musculoaponeurotic weakness allows the surgeon to align the operation with the true source of deformity, whether this means isolated dermolipectomy, formal abdominoplasty with plication, or broader reconstructive strategies in post-weight loss patients. This approach is directly applicable to daily plastic surgery practice across public referral hospitals and private tertiary centers in Mexico (including Instituto de Seguridad y Servicios Sociales de los Trabajadores del Estado (ISSSTE)-based practices and ABC Medical Center) as well as to international aesthetic and reconstructive settings, where reproducibility, patient selection, and comparability of outcomes remain essential. Ultimately, future research should focus on standardizing diagnostic thresholds, imaging protocols, functional endpoints, and patient-reported outcomes so that abdominal contour surgery can move from heterogeneous technical preference toward more consistent, evidence-based decision-making.
